# Exploring individual and work organizational peculiarities of working in emergency medical communication centers in Norway- a qualitative study

**DOI:** 10.1186/s12913-019-4370-0

**Published:** 2019-08-02

**Authors:** Ann-Chatrin Leonardsen, Helge Ramsdal, Theresa M. Olasveengen, Jon E. Steen-Hansen, Fredrik Westmark, Andreas E. Hansen, Camilla Hardeland

**Affiliations:** 1 Department of Health and Welfare, Ostfold University College, Postal box code (PB) 700, NO-1757 Halden, Norway; 2 Ostfold Hospital Trust, Surgical Ward, PB 300, NO-1714 Sarpsborg, Norway; 3 Department of Health and Social Studies, Ostfold University College, PB 700, NO-1757 Halden, Norway; 4 Department of Anaesthesiology, Oslo University Hospital, PB 4956, NO-0424 Nydalen, Oslo Norway; 5 Vestfold Hospital Trust, Prehospital Clinic, PB 2168, NO-3103 Tønsberg, Norway; 6 Ostfold HF Hospital Trust, Prehospital Clinic, PB 300, NO-1714 Sarpsborg, Norway; 7 Prehospital clinic, Oslo University Hospital, PB 4956, NO-0424 Nydalen, Oslo Norway

**Keywords:** Emergency medical dispatch, Emergency medical communication centers, Core technologies, Decision making, Call taker, Emergency medical dispatcher, Management, 1-1-3-calls, 9-1-1- calls, Situation awareness

## Abstract

**Background:**

Emergency Medical call-takers working in Emergency Medical Communication Centers (EMCCs) are addressing complex and potentially life threatening problems. The call-takers have to make fast decisions, responding to problems described in phone calls. Recent studies focus mainly on individual aspects of call-takers’ work. The objectives of this study were to explore 1) What characterizes individual work performance of call takers in EMCCs? and 2) What characterizes work organizational factors call takers see as most relevant to the performance of their work?

**Methods:**

The research is based upon in-depth interviews with call takers at three EMCCs in Norway (*n* = 19). Interviews were performed during the period May 2013 to September 2014. Data was analyzed using thematic analysis.

**Results:**

Two main themes that related to individual work performance and to work organizational factors in EMCCs were identified, namely: 1) “Core technologies” and 2) “Environmental issues” . The theme “Core technologies” included the subthemes a) multiple tasks, b) critical incidents, and c) unpredictability. The theme “Environmental issues” included the subthemes a) lack of support, b) lack of resources, c) exposure to complaints, and d) an invisible service.

**Conclusion:**

At the individual level, multiple tasks, how to cope with critical incidents, and the unpredictability of daily work when calls are received, make the work of call takers both stressful and challenging. The individual call taker’s ability to interprete the situation by intuition and experience when calls are received, is the main factor behind the peculiarities working in the centers at the individual level. At the organizational level, the lack of resources and managerial support seems to provoke concerns about the quality of services rendered by the centers. These aspects should be taken into account in the managing of these services, making them a more integrated part of the health service system.

**Electronic supplementary material:**

The online version of this article (10.1186/s12913-019-4370-0) contains supplementary material, which is available to authorized users.

## Background

In cases of medical urgency, emergency medical communication centers (EMCCs) are the primary contact point between callers and the health care system. The EMCCs are manned with emergency medical call takers responsible for answering emergency calls, assessing the need for health care (triage), mobilizing necessary resources and providing pre-arrival instructions to ensure lifesaving first aid until the ambulance arrive [1]. In most nordic countries call taking has become increasingly performed by registered nurses [2]. In addition, the EMCCs are staffed by emergency medical technicians (EMTs), or – dispatchers (EMDs) dispatching ambulances.

The organization of EMCCs varies substantially throughout the world, and includes a diversity of combinations of ambulance personnel, paramedics, specialized nurses,, emergency physicians and physicians [3, 4]. In Norway, the EMCCs are normally located at a regional hospital, although co-localisation with the other emergency servicesalso exists. In addition to the public 1-1-3-line, the centers also receive direct lines from out-of-hours emergency primary health care, in-hospital emergency lines, ambulance booking lines, and admission lines from General Practitioners (GPs).

In the case of call takers’ work, this expertise is both related to professional knowledge, and formalized by protocols (e.g. the Norwegian Index for Medical Emergencies [5]). Devoid of visual and physical contact with the patient, the call taker is required to remotely provide services, using their verbal and audio skills to locate the patient, identify and classify the medical condition, employ and coordinate appropriate resources, alerting the appropriate patient destination within the health services, and provide critical first aid instruction whilst mediating stress and trauma of the caller [6]. The work is characteriszed by multiple tasks, critical incidents, and unpredictability, making it potentially one of the most stressful work in public services [7].

Studies on EMCCs have mainly focused on individual aspects of call takers’ work. We wanted to further explore these aspects, but also to include organizational factors impacting on the work of call takers. As healthcare personnel with experience both as call takers, dispatchers, registered nurses and medical doctors, as well as a professor in political science/organization theory, our objectives were to explore

- What characterizes individual work performance of call takers in EMCCs?
- What characterizes work organizational factors call takers see as most relevant to the performance of their work?

## Materials and methods

We used a qualitative approach, conducting individual in-depth interviews with call takers to explore individual and organizational peculiarities of their work.

### Setting and participants

In Norway, there are 16 EMCCs (2019), providing services to all 5.2 million inhabitants. Organization and function of the EMCCs are regulated by law [8], but EMCCs have some differences and challenges due to the diversity of population density and topography. Some EMCCs cover large, rural areas with low population and a topography including e.g. mountains and coastline. Other EMCCs cover smaller, urban areas, or one EMCC can cover all of the above. Maximum variation purposive sampling was captured by including EMCCs from both rural and urban areas serving both smaller and a large population [9]. The study was conducted in three different EMCCs in the Southern part of Norway:

EMCC 1 covered three regions, consisting of both rural and urban areas and a population of approximately 1,2 million people. In 2013, EMCC 1 received approximately 315,000 calls of which approximately 124,000 were emergency calls. The region had 45 regular ambulances at its’ disposal in addition to one single paramedic manned ambulance, one motorcycle unit, and one physician staffed rapid response vehicle. In addition, EMCC 1 was responsible for two physician staffed helicopters. The center employed 25 emergency medical technicians (EMTs) coordinating ambulance responses and 29 registered nurses answering emergency calls.

EMCC 2 served a population of approximately 400,000 people, and deployed 31 ambulances. It was staffed by registered nurses with additional training in emergency medical dispatch answering emergency calls, and EMTs coordinating ambulance responses. In 2013, they responded to 32,776 emergency calls and handled a total of 63,025 calls.

EMCC 3 covered a population of 287, 000 people. It deployed 23 ambulances, and was staffed by registered nurses with additional EMCC training answering emergency calls, and EMTs coordinating ambulance responses. EMCC 3 employed 21 dispatchers, handling almost 110,000 calls per year, responding to approximately 42,000 calls of which approximately 28,000 are emergency calls.

In the three EMCCs, we used a purposive sampling method, selecting information rich cases for in-depth study. The interviews are part of a larger study on EMCCs and how they handle cardiac arrest cases, hence, we had access to and reviewed audio files (*n* = 1095) of all calls concerning cardiac arrest in the three study sites during the study period [9]. Cardiac arrest is an event that require high level of skills by the dispatchers, with rapid decision-making in a process where every second counts. Dispatchers need to recognize that it is actually a cardiac arrest, dispatch appropriate resources and provide instructions on cardiopulmonary resuscitation to lay people in order to save lives. Cases were selected based on calls representing both successful decision-making, and calls containing barriers and challenges. Corresponding call takers were invited by mail to participate in an interview.

### Datacollection

A semi-structured interview guide was developed over several iterations, and was based on existing literature as well as discussions among the authors (Additional file 1). The guide focused on the participants’ social and physical work conditions, work load, organizational factors and management, use of relevant decision support tools, and how to handle emergency calls in general. The interviewer focused on identifying the participants’ experiences, and how these experiences were presented. Moreover, participants were continuously asked what they really meant during the interviews, and their statements were mirrored with the interviewer asking for correction or approval to verify that the participant had been interpreted correctly.

The interviews were conducted by the last author in an office in proximity, but separated from, the EMCC, during the call takers’ working day. Reflexive practice, including the scrutiny of the researcers own impressions, positioning and emotional investments, was applied throughout the data collection, as well as during the analysis [10, 11]. After each interview, the last author wrote down initial impressions and thoughts from the interview. During the analysis analytic memos, including possible discourses, impressions and experiences that could possibly impact the interpretations, were written down. Interviews were continued until theme saturation, indicated by data replication and the identification of no new themes [12], was reached. All interviews were digitally recorded and transcribed verbatim, and had a duriation of from 40 to 90 min.

Interviews were performed during the period May 2013 to September 2014. A total of 19 call takers agreed to participate in the study, and were consecutively interviewed.

### Analysis

Data was analysed using thematic analysis as recommended by Braun and Clarke [13], including five steps identifying, analyzing and reporting themes within the data (Table 1).

**Table 1:** Thematic analysis as recommended by Braun and Clarke (2006)

Step 1: Familiarisation	Step 2: Coding	Step 3: Identifying themes	Step 4: Naming overarching themes	Step 5: Reporting
Reading and re-reading the data, noting down initial ideas	Manually, inductive data-driven coding of the data	Collating codes into potential themes	Reviewing themes Overall interpretation of the data	Producing the report

Abbreviation: the process was a recursive, iterative process moving back and forth throughout the steps

First, each individual interview transcript was coded inductively in Norwegian by the first author. The coding identified the most basic elements of the data that carried meaning. These codes were discussed until agreement between the first and the last author was reached. Counting of codes across all of the interviews lead to the recognition of patterns in the data, and deviations from those patterns [14]. The codes, themes and the final analysis were then discussed and interpreted as an iterative, recursive process, until consensus was achieved by all of the authors. Discussions also included an evaluation of whether the findings were shaped by the participants and not as bias due to the researchers’ motivation or interest. Reflexivity was presented as the researchers, when evolving the results, continuously revisited the data, bringing in the developing comprehension of it in light of the revised understanding of different aspects of the topics throughout the analysis [10, 15]. This included a focus on the researchers’ personal and professional experience, cultural factors, assumptions and hunches that could influence the interpretation of the data (e.g. one of the researchers has worked in an EMCC previously). An example of the analysis process from one of the interviews is presented in Table 2.

**Table 2:** Analysis process- from codes to overarching themes

Code	Theme	Overarching theme	Quote
Multitasking Talking to the caller Talking to the EMT Prioritizing Assessment Evaluation Comfort the caller Give advice	Multiple tasks	Individual work performance	*“After I started instructioning the caller, I tried to get in contact with him (the EMT), to see if there were any ambulances available”*

## Results

From EMCC 1 ten call takers participated, while five call takers participated from EMCC 2 and four call takers from EMCC 3. The call takers had been working in an EMCC for an average of seven years (range 0–19 years), and had an average of 12.6 years of working experience as a nurse (*n* = 17)/ paramedic (*n* = 2). Mean age was 45.2 years (range 29–62 years), 21% male.

Two main themes were identified, namely: 1) Core technologies and 2) Environmental issues. The theme “Core technologies” included the subthemes a) multiple tasks, b) critical incidents, and c) unpredictability. The theme “Environmental issues” included the subthemes a) lack of support, b) lack of resources, c) exposure to complaints, and d) an invisible service.

### Core technologies

The call takers identified several issues that were related to the core technologies in their individual work performance.

#### Multiple tasks

The participants described their work as very complex. Responding to the 1–1-3-calls included not only to talk to the caller, but also to verify where the patient was, to try to identify the problem and to decide how to respond, as well as to give the caller instructions about what to do until the help arrived. In addition the job included to be in contact with the EMT coordinating the ambulances, as well as to respond to additional phonecalls from e.g. General Practitioners (GPs). In addition they had limited time to consider the options.

Most of the participants described to do their triage based on earlier experiences, since they had no visual or physical connection with the actual patient. Participant 13 expressed: “I use my experience and intuition. This makes me able to ask further, because I have the ballast I do”.

The participants were also aware of the tone of the callers voice, and signs of stress or anxiety. They also considered the patients’ age and normal vital parameters, and even location and time: “It depends on how close it is. We may not have any ambulances nearby” (Participant 18), and: “What was he doing up at that time at night?” (Participant 14).

Call takers used words like “perceive”, “experience”, “interpret”, “feel”, “hear” or “understand” when describing their own decision-making. Participants emphasised that there were seldom doubt in critical cases, and that it was more difficult to judge in more vague situations.

The participants identified several other tasks during the 1–1-3 calls, depicted as to “comfort the caller”, “build trust” or “support and help”. Communication challenges due to language difficulties were common, as described by many of the call takers.

#### Critical incidents

Many of the participants described calls that had made great impact upon them, especially related to incidents involving children. The participants described calls relating to “life or death”, and callers in crisis. Many of the participants described this a psychological challenge. Participant 10 stated: “Obviously, sometimes you feel that this case is lost. I suppose it is, but I cannot be sure”.

The work at the EMCC exposed the participants to a great variation of events, being the first person in contact with the involved caller. Calls ranged from non-acute medical problems that should have been directedelsewhere, to critical accidents, severe illness and suicides. The calls made great influence on the call taker, as described by e.g. Participant 18: “It’s the psychological strain to sit there, knowing that no matter how large the incidents, I’m alone with it”. This was supported by e.g. Participant 12 who stated: “… getting the dispair straight into my ear, into my head, and it won’t disappear ”.

Several of the participants told that after a critical incident with many impressions, they needed a time-out, not having to answer the next 1-1-3 call for a while.

#### Unpredictability

The only thing the participants could prepare for was to come to work, sit down and start taking the phonecalls. The rest was up to the caller, or what happened outside in the society. Participant 16, who had only two months experience from the EMCC, said: “Well, I’m not like adrenaline-kick-prepared. But I always have a mentality that I have to be ready for worst case scenarios”.

This was also emphasized by more experienced call takers, e.g. Participant 17, who stated: “You never know if it’s an ordinary chest-pain call, or whether a plane has crashed”. Moreover, participant 1 expressed: “We are feeling stressed all the time. Even though we are not physically active. It’s not a normal physiological situation- we sit still and are stressed out”.

Due to this unpredictability, several of the recruits to new jobs at the EMCCs quitted after the training period.

### Environmental issues

The participants also identified several organizational factors that were relevant to their work performance.

#### Lack of support

Immediately after a critical incident some of the participants described a need to “take a time-out”, leave the phone and take a walk, finding themselves “just staring into the screen in front of me” (Participant 15). Unfortunately, this was not always possible, due to multiple incoming 1-1-3- calls. Structured debriefs were performed amongst ambulance personnel, but call takers were seldom invited to these kind of events. Most of the call takers drew a picture of an absent management. This implicated that the call takers did not get any indication on the quality of their own performance, as Participant 19 stated: “I do not receive any feedback. If I choose to make a bad decision, there’s nobody there to get me on the right track”.

Most of the call takers also emphasized that, if they eventually heard something from their leader, it was in cases of an error or a complaint. Call takers often experienced being called by managers at home, as described by Participant 14: “What’s not okay is that you receive a call, in your spare-time… I could have been sleeping, having a vacation, a day off. This destroys my whole day”. This aspect led to that call takers asked multiple questions to clarify most situations to “secure their own back” (Participant 6), because they did not feel sure that their leaders would support them if needed.

#### Lack of resources

Lack of resources where both related to internal as well as external resources. Internal resources where defined as technical equipment such as head-sets that were not able to shut out disturbing noices from the central, not functionable map-systems or computer systems that did not communicate with each other. This also included a lack of call takers to respond to the 1-1-3-calls, and EMTs to coordinate the ambulances. This was especially visible during evening and night shifts, as well as in weekends. In all of the EMCCs leaders stribed to reduce answering time, and confronted call takers with delays. Participant 13 claimed: “It is not bearable! When they (the managers) point out that four per cent of the calls waits for 20 seconds, and that is too much”.

According to this call taker, this had an impact on each phonecall: “This certainly affects us and how we end the conversation in each call, right”.

Lack of personnel also implicated that there was not room for any breaks if a call taker needed a “time-out”, or even to eat or to visit the toilet.

In means of external resources this was related to a lack of ambulances, which again led to frustrations and arguments between call takers and EMTs. Due to this, call takers felt a somewhat pressure not to send ambulances. But, as Participant 5 prompted: “I cannot set another criterion just because we don’t have enough vehicles”.

The availability to both internal and external resources negatively affected the EMCC personnel and their experience of their own job. Participant 19 stated: “You are multi-tasking thoughts and worries at work when there are few vehicles, and a major accident occurs. And then we have no guidelines in what to do, in cases of chaos”.

Finally, the lack of resources had an impact on the opportunities for professional development. Several of the participants told that compensatory days off were withdrawn due to this.

#### Exposure to complaints

Call takers also expressed to be exposed to a lot of complaints at work. This occured both during a day at work, and as written complaints sent to management or health authorities. At work, primarily, this was from callers, who felt that they did not receive the help they expected. Call takers described callers who were “preachy”, “yelling” or “threathening”.

Callers were described as in a crisis, they were scared, desperate, felt unsecure. Nevertheless, this reaction was experienced as a burden by the call takers. It also made it more difficult to do a triage, more difficult to divide reaction and actual problem or situation: “It’s difficult to know whether he fell down the stairs. Is he drunk? Is he awake? Is he hurt?” (Participant 10).

In addition, call takers received complaints from hospital wards, GPs or primary health care providers, who had to wait for the ambulance, leading to patients not being sent home or to appropriate level of care or not being in time for e.g. specialist appointments. Most of the call takers felt pressured by these complaints. Some had negative experiences from cases brought forward to the Norwegian Board of Health Supervision, negative focus in the media, and not being allowed to answer to the complaints, due to confidenciality. According to several of the participants, these experiences, or rumours, made call takers send out more ambulances than they actually judged necessary. This again led to complaints from the ambulance personnel, as well as from the EMTs.

#### An “invisible service”

Finally, call takers described their job as “an invisible service” or a “secret service”, because neither healthcare personnel nor the public seemed to know or understand the contents of it, or the difference they could make. The EMCCs were located in separate buildings, and personnel had to use key cards to get in there. Participant 13 stated: “This important society service. If we are put out, it will hit 1.2 million people. In this little bubble we are saving lives, right”.

The “saving lives” experience was shared by most of the call takers, and seemed to be a great motivation to keep such a challenging job. Nevertheless, several of the call takers would have appreciated positive feedback, as emphasized by Participant 3: “People have no idea what kind of competence we have. We get no acknowledgements for the work we do, or the competence we have”.

Due to that neither the public nor healthcare personnel had this knowledge, the EMCCs were wrongly used; e.g. people called them instead of their GP, which increased the pressure on the call takers. Participant 4 stated: “You have to accept much negativity, and then you get little positivity back. The weighing scale do not reach a balance. And then we don’t even get to see the one you are helping”.

Call takers emphasized the difference in acknowledgement between clinical work and their own, as Participant 14 stated: “The ambulance personnel get his kind of acknowledgement- they are the ones saving lives”. But, she continued: “I think you have to be sort of proud of what you do. Think that we are some kind of heroes”.

This approach seemed to be common amongst all of the call takers- that they were proud of their job.

## Discussion

The call takers characterization of individual work performance related to multiple tasks, critical incidents and unpredictability. Lack of support from management, lack of resources, exposure to complaints and a feeling of invisiblity were organizational factors call takers’ reported made an impact on their work performance.

The multiple tasks that characterized the call takers work can be referred to as the EMCC core techonolgy- the primary function the EMCC performs at that defines the “production process” in which it engages [16]. In the EMCC this includes the transformation of emergency calls to prioritization and decisions. This diversity has also been described in other studies (e.g. [7, 17]). Ek and Svedlund [2] found that EMCC call takers reported difficulties conveying medical advice without seeing the patients. Even so, they found their work to be stimulating and full of opportunities, as indicated in the current study.

Many of the participants described calls that had made great impact upon them, and they described this a psychological challenge. Stressors identified in a recent study included being exposed to traumatic calls, lacking control, and working in stressful environments [7]. Unclear circumstances have been shown to increase stress level, with cases involving children being especially stressful [18], as described in our study. Most of the participants emphasized the unpreparedness in their work situation, which further increased the experience of stress. Having discussions with colleagues directly after the assignment have been shown to reduce stress [7, 18]. This was something the participants reported missing. Demands in the workplace, levels of perceived control and support by the organization are fundamental elements that can moderate workplace wellbeing [19].

Most of the call takers described an abscent management, not giving any feedback or support. Research support this, showing that emergency personnel feel frustrated due to poor management, and this enhance the burden of professional responsibility and increase reported stress [7, 17, 20, 21]. Hence, management have an essential role in decreasing the EMCC personnels’ feeling of stress and extensive work demands through being visible, and facilitate for personnel to discuss situations. Organizational systems for feedback in EMCCs are sparse, and the most utilized source of feedback is complaints from the public and authorities. These feedbacks are always negative and does not contribute to a positive work environment. Much of the patient safety improvement literature calls for moving away from a punishment-governed culture of blame to a more empathic, interdependent, and positive context. A healthcare culture of interpersonal trust, success seeking, and positive behavior change is needed [22]. It would be helpful for EMCC management to have access to organizational systems for feedback to be able to identify succesful calls in order to give positive feedback on a regular basis.

When call takers were involved in critical incidents they had a need for a “time-out” to reflect on what had happened. These needs were not met. Research has shown that call takers who are invited to structured debriefs perceive this as management acknowledging the importance of their role in the organization. Conversely, exclusion from this has been found to contribute to a sense of decreased value by the organization [23]. Research indicate that debriefing has been successfully used in resuscitation and critical care settings, as well as an educational strategy to improve clinician skill acquisition [24–26]. Hence, inviting EMCC personnel to debriefs after critical incidents could decrease their experience of a stressful work situation.

Moreover, lack of resources affected the call takers’ work performance. Problems with technical outfit could have reduced response time if improved. Lack of ambulances available sometimes caused conflicts with EMTs. who had to distribute ambulances at a regional level. Here, there is a potential conflict between the call takers’ professional judgements when receiving calls, and the overall planning of allocation of ambulance resources [2, 17]. This is an important issue that should be presented at a political level to emphasize the need to increase resources to increase patient safety.

Call takers expressed a need for a more comprehensive quality improvement strategy. E.g. Ek and Svedlund [2] emphasize the need for a blame-free culture as an important factor to attract and retain employees. Research has identified how experiences of negative interactions from both EMTs and management can uphold perceptions of call takers that they are undervalued by the organization [23]. Moreover, the impression was that the EMCC services were an invisible service, and that people at large knew very little about working in the EMCC. This “invisibility” of emergency centrals has also been described in other studies [17, 21, 27]. Hence, management plays a central part in focusing on positive feed-back, establishing a blame-free culture, and focus on quality improvement strategies. In 2018–19 a show called “1-1-3” has been sent on Norwegian television, presenting real-life situations of critical illness, following and filming the work of personnel in the EMCC, ambulance and hospital. This may have increased the public acknowledgement of the EMCC work.

Both individual and work organizational factors can be seen as part of situation awareness (SA), linked to call takers’ experiences in addition to their professional knowledge. SA has been described as a state of knowledge, and an umbrella term for a range of cognitive processes, or as a skill [28, 29]. Cooper et al. [30] claims that SA is the call taker’s “internal model of the environment from which they can decide what actions to take”. Three levels of SA have been described: perception, understanding and prediction [31]. E.g. Wong & Blanford emphasized that senior dispatchers use SA to plan ahead and to evaluate what had been done [32].

The call takers referred to a “mental picture” of what was going on at the locality, as supported by other studies [30]. The “mental picture” of the situation at the site had to be combined with considerations about professional guidelines of triage, but also the availability of ambulances, the distance to hospitals, and how to cope with the individual call when other emergency calls are at wait. Here, the peculiarities of this kind of intensive work sometimes is referred to as using “experience and intuition”. Studies indicate that higher levels of SA are exhibited by call takers for non-routine or major incidents than for routine incidents [32]. Conversely, in our study call takers reported to use the medical index more often in major incidents such as cardiac arrests, than in routine incidents. Internationally, it has been recognized that failures in non-technical skills, such as SA, contribute to complications and adverse events [33–35]. Specific initiatives have been launched internationally to increase the training in such skills [35–37]. Hence, focusing on SA in simulations or training of EMCC personnel could be useful to improve the quality of their work.

Research on the EMCCs have focused mainly on SA of individual call takers, not addressing the work organization in general. The complex relations between the characteristics of SA and other work organizational characteristics; e.g. financial resources, organizational design, or management, need to be addressed in order to understand the way the call takers work in a more comprehensive way. Consequently, our findings fill a knowledge gap, including both individual and work organizational aspects of SA in EMCCs to better understand and improve this skill.

### Limitations

The EMCCs were organized differently, and this may potentially impact on the findings. In addition, data was gathered in 2013 and 2014, and the EMCCs have been through several structural changes since then. Hence, our findings reflect the call takers’ perspectives at that time.

Since sampling were made based on both successful and challenging 1–1-3-calls, the nature of the calls could have impacted the findings. Nevertheless, this aspect was included during the process of analysis.

The transparency in the methods description indicate valid findings, also indicated through issues of credibility, dependability, confirmability, and transferability.

Credibility includes a confidence in the “truth” of the findings [37]. In the current study, credibility was achieved by providing a thorough description of the data collection, as well as the steps of the thematic analysis. Relevant literature was used when developing the research questions and interview guide, as well as when interpreting the findings. Letting the participants read through the transcripts could have increased the credibility of the study, but this was not done.

Using a digital recorder, as well as rigorous and thorough orthographic transcripts including verbatim accounts of all verbal and nonverbal utterances, ensured dependability. This also support that findings are consistent, and that the study could be repeated.

Confirmability was achieved through discussion of the codes, subthemes and themes among the researchers until agreement was reached. In the qualitative research design lies the lack of opportunity for generalisation of findings. Even though it could seem like the issues highlighted here are solely of a national concern, findings are in-line with findings from other countries. Transferability was sought through the inclusion of participants from different EMCCs, of both gender and a range in both age and years of experience.

Reporting of findings are in-line with the Consolidated criteria for reporting qualitative studies (COREQ) [38].

## Conclusions

At the individual level, multiple tasks, how to cope with critical incidents, and the unpredictability of daily work when calls are received, make the work of call takers both stressful and challenging. The individual call taker’s ability to interprete the situation when calls are received by intuition and experience, is the main factor behind the peculiarities working in the EMCCs at the individual level. At the organizational level, the lack of resources and managerial support seems to provoke concerns about the quality of services rendered by the centers. These aspects should be taken into account in the managing of these services, making them a more integrated part of the health service system.

## Additional file

Additional file 1: Interview guide. (DOCX 16 kb)

## Data Availability

The datasets generated and/or analysed during the current study are not publicly available due to local ownership of the data, but are available from the corresponding author on reasonable request.
