# Supplementary material for: Exploring individual and work organizational peculiarities of working in emergency medical communication centers in Norway- a qualitative study
Source: BMC Health Serv Res. 2019 Aug 2;19:545. doi: 10.1186/s12913-019-4370-0 (PMC6679546; doi:10.1186/s12913-019-4370-0)
Supplement: Supplementary file 1 — Interview guide. (DOCX 16 kb) [file 12913_2019_4370_MOESM1_ESM.docx]

**Additional file 1**

**Interview guide**

*1.Background*

Information about the project and themes of the interview. Participants age, education, work related experience.

*2. Critical incident*

- Can You please tell me about the incident?
- How did You experience the communication with the caller?
- How did You experience the communication with the dispatcher, or other colleagues involved?
- Were there any special circumstances that had an impact on the incident?
- I would now like to play the audio file of the call if that is okay? You may comment during this sequence.
- (after playing the audio file): How did You experience listening to this?
- What are Your thoughts now, after hearing this? Are You aware of something else now, that may have affected You?
- How do You make decisions related to agonal breathing?
- If You could take this call over again, would You do anything different?(What?)
- Do You want to emphasize anything that worked particularly well during the call?
- What is Your overall impression of your handling of this call?
- How do You assess the situation if the caller says the person is dead?

*3. The Norwegian Index for Emergency Medical Assistance (The index)*

- How well do You know the guidelines for handling cardiac arrest as described in the medical index?
- How do You experience the index works for you in general as a decision-support tool at the EMCC?
- What are Your thoughts about the use of the index in a cardiac arrest situation?
- Did You use the index during this particular call?
- If You did- which part did You use? Do You have any thoughts about why/why not You used the index?
- Do You use the index for triage or for instructions to the caller?
- Do You have any suggestions for any improvements of the index?

*4. Context*

- Could You please describe a normal day at the EMCC?
- Can You tell me a bit about the working conditions?
- Can You tell me something about sharing of tasks / distribution of work? How is the EMCC manned?
- Do You have any impact on Your own work situation?
- How do You experience support from Your nearest manager?
- How are potential conflicts or disagreements solved?
- To which extent do You feel that the overall organization of the EMCC have an impact on Your work conditions?
- What were the circumstances that actual day in question?
- Did anything else happen in the EMCC that had an impact on the call?
- How long do You plan to continue working in the EMCC? At any time, have You had plans to quit your work at the EMCC?

Thank You for participating.
